# Recognition memory for pictorial material in subclinical depression

**DOI:** 10.1016/j.actpsy.2010.07.015

**Published:** 2010-11

**Authors:** Cristina Ramponi, Fionnuala C. Murphy, Andrew J. Calder, Philip J. Barnard

**Affiliations:** Medical Research Council, Cognition and Brain Sciences Unit, Cambridge, UK

**Keywords:** Depression, Memory, Emotion, Recollection, Familiarity

## Abstract

Depression has been associated with impaired recollection of episodic details in tests of recognition memory that use verbal material. In two experiments, the remember/know procedure was employed to investigate the effects of dysphoric mood on recognition memory for pictorial materials that may not be subject to the same processing limitations found for verbal materials in depression. In Experiment 1, where the recognition test took place two weeks after encoding, subclinically depressed participants reported fewer know judgements which were likely to be at least partly due to a remember-to-know shift. Although pictures were accompanied by negative or neutral captions at encoding, no effect of captions on recognition memory was observed. In Experiment 2, where the recognition test occurred soon after viewing the pictures, subclinically depressed participants reported fewer remember judgements. All participants reported more remember judgements for pictures of emotionally negative content than pictures of neutral content. Together, these findings demonstrate that recognition memory for pictorial stimuli is compromised in dysphoric individuals in a way that is consistent with a recollection deficit for episodic detail and also reminiscent of that previously reported for verbal materials. These findings contribute to our developing understanding of how mood and memory interact.

## 1 Dysphoric mood and its effects on memory for pictorial material

Depression is associated with impaired memory function. Previous findings have indicated that dysphoric mood specifically impairs recognition memory accompanied by the *recollection* of the encoding context in which material is first seen, and not recognition memory that is based in *familiarity* (Drakeford et al., 2010; Hertel & Milan, 1994; Jermann, Van der Linden, Adam, Ceschi, & Perroud, 2005; MacQueen et al., 2003; MacQueen, Galway, Hay, Young, & Joffe, 2002; Ramponi, Barnard, & Nimmo-Smith, 2004; see also Lemogne et al., 2006; Raes et al., 2006). Studies such as these have narrowed down the memory impairment associated with depression to the recollection component of recognition memory. This finding is of great significance as therapeutic interventions could exploit the spare, more automatic, capacity of familiarity-based recognition. Nevertheless, the recollection deficit remains poorly understood and replication has not been universal (see, Jermann, Van Der Linden, Laurençon, & Schmitt, 2009). In the current research, we investigated the effects of subclinical depression on the familiarity and recollection components of recognition memory for pictorial material as this allowed us to explore the important role that *visual* cognition plays in depressive ideation.

Visual processing, compared to verbal processing, is differentially influenced by depression, thus memory for pictorial material may differ substantially from memory for verbal material. For example, Baker and Jessup (1980) argue that verbal processing is more characteristic of depressive ideation than visual processing; when dysphoric participants were directed to process information visually (with mental images), rather than verbally, visual processing was rated as more euphoric than verbal processing. Bywaters, Andrade, and Turpin (2004) reported a positive correlation between sad mood and vivid imagery when recalling pictures, suggesting that visual processing was less susceptible than verbal processing to disruption in depression. The processing of images is likely to make use of resources that do not completely overlap with those required for verbal ideation as proposed in different models of working memory (Baddeley, 2007; Barnard, 1999). Image processing may thus distract from, rather than prompt, classic abstract depressogenic themes such as “failure.” On the basis of these kinds of evidence, it has been suggested (see, Holmes, Arntz, & Smucker, 2007) that mental imagery could be used in cognitive behaviour therapy in order to alleviate negative emotional symptoms. Yet, basic knowledge of the effects of sad mood on recognition memory for pictorial material remains sparse.

Pictures are informationally richer than words: whereas the word “telephone” covers a multitude of physical forms, a picture of a phone depicts a specific model of phone with some clearly delineated attributes and hence offers greater potential for recollection. In fact, memory for pictures is usually better than memory for words — the *picture superiority effect*. Dewhurst and Conway (1994) argued that pictures give rise more readily to an enriched automatic recollective experience because the encoding experience can be richer both perceptually and semantically. Dewhurst and Conway (1994) and Rajaram (1996) have demonstrated that in recognition memory, the picture superiority effect is confined to the recollection component by using the remember/know procedure (Gardiner, 1988; Tulving, 1985). After recognising a picture or a word as seen before, participants were asked to indicate whether they could recollect the encoding context, in which case they assigned a “remember” judgement, or whether they simply knew that they had come across the item before but could not recollect the context in which the item was first seen, (i.e. the item was familiar) in which case they assigned a “know” judgement. More remember judgements were reported for pictures than for words, whereas an equal or larger number of know judgements were reported for words relative to pictures. It has also been shown that different brain regions are involved in the recollection of pictures and words (Kensinger & Schacter, 2006; Woodruff, Johnson, Uncapher, & Rugg, 2005).

There is increasing evidence that the memory difficulties experienced in depression are better characterised as an impairment of recollection and not familiarity (Drakeford et al., 2010; Hertel & Milan, 1994; Jermann et al., 2005; MacQueen et al., 2002, 2003; Ramponi et al., 2004). Depressed individuals when recognising items presented previously, have little difficulty with realising that the item was seen before, but they have considerable difficulty remembering the item spatial or temporal context. More specifically, with the remember/know procedure, it was found that recognition responses assigned with remember judgements were reliably fewer in depressed (Drakeford et al., 2010) and subclinically depressed participants (Ramponi et al., 2004) than in controls, whereas the number of recognition responses assigned with know judgements did not differ reliably. The first goal of the present investigation was to determine whether the same pattern of impaired recollection and intact familiarity would be observed for pictorial stimuli. Given the arguments just exposed that dysphoric mood affects verbal, but not visual, processing, it was possible that spared visual processing capacity could offset any recollection deficit.

The second goal was to study the modulatory effect of the emotional content of memoranda on the recollection deficit. Emotional salience is known to have a very strong effect on memory for events (for reviews, see Buchanan, 2007; Hamann, 2001; Kensinger, 2004; LaBar & Cabeza, 2006). As life experiences worth remembering must be discriminated from experiences that can be forgotten without cost, emotional salience is a clear candidate for marking out particular experiences for privileged mnemonic access. The memory enhancing effect of emotion has been observed across a number of paradigms and for different events or stimulus types. There is also substantial evidence that emotional stimuli are better *recollected* than neutral ones (Dewhurst & Parry, 2000; Kensinger & Corkin, 2003) and that this holds for pictorial stimuli as well (Comblain, D'Argembeau, Van der Linden, & Aldenhoff, 2004; Dahl, Johansson, & Allwood, 2006; Dolcos, LaBar, & Cabeza, 2005; Ochsner, 2000; Sharot, Delgado, & Phelps, 2004; Sharot & Yonelinas, 2008; but see Aupee, 2007) and also that the emotional context in which to-be remembered items are appraised influences memory. Pre-experimentally neutral objects or words that are embedded in emotional pictures are better recalled than those embedded in neutral pictures (Erk et al., 2003) and show different neural activation patterns at retrieval (Smith, Henson, Rugg, & Dolan, 2005). Similar effects have been reported for the retrieval of neutral words (Maratos, Dolan, Morris, Henson, & Rugg, 2001) and pictures (Cahill & McGaugh, 1995) when the emotional context is determined by accompanying prose.

In depression, the memory enhancement effect for negative emotional material is even more pronounced. Depression has been linked with an abnormal appraisal of emotional stimuli (for a review see Leppanen, 2006) that contributes to the onset and maintenance of depression. This distorted emotional information processing is reflected in enhanced memory for negative emotional material, i.e. material congruent with sad mood (Blaney, 1986; Elliott, Rubinsztein, Sahakian, & Dolan, 2002; Murphy et al., 1999). Cognitively, this effect is thought to be mediated by increased allocation of processing resources to negative material as more elaborate associations are generated to information consistent with an individual's current concerns (see Blaney, 1986; Williams, Watts, MacLeod, & Mathews, 1997). Neuroimaging investigations suggest that the abnormal amygdala activation typically associated with sad mood (for a review see Drevets, 2003) may mediate the memory enhancement of negative material (Moritz, Gläscher, & Brassen, 2005).

The memory enhancing effect for negative material in depressed individuals has been observed for faces that vary in emotional expression (Gilboa-Schechtman, Erhard-Weiss, & Jeczemien, 2002; Ridout, Astell, Reid, Glen, & O'Carroll, 2003; Ridout, Noreen, & Johal, 2009) and Jermann, Van der Linden, and D'Argembeau (2008) observed that this recognition memory enhancement was captured specifically by the recollection component, as is the case for words (Jermann et al., 2009; Lewis, Critchley, Smith, & Dolan, 2005). Whether pictures of scenes that have negative emotional connotations are also better retained by participants experiencing dysphoric mood, potentially offsetting the general recollection impairment remains unclear (for example see Jermann et al., 2009). This question was addressed in the current research by comparing dysphoric and control participants on recognition memory for pictures varying on an emotional dimension. In the present investigation, we tested, first, whether subclinically depressed participants demonstrated impaired recollection and intact familiarity for pictures, and second, whether emotion can modulate picture memory.

## 2 Experiment 1

Experiment 1 evaluated the effect of mood on recollection for neutral pictures that did not convey inherent affective information. It was possible that a recollection deficit parallel to that found for verbal material (Drakeford et al., 2010; Ramponi et al., 2004) would be observed, although any deficit could be offset by a relative preserved visual processing capacity.

The remember/know procedure was employed to explore which component of picture recognition, if any, is affected by dysphoric mood. Contrary to previous protocol for verbal materials, we followed the precedent set by Ochsner (2000) for pictorial materials so that ceiling effect could be avoided and did not test memory immediately, but after a two-week delay. With this significant delay between study and test, a recollection deficit can also be expected to be evident as a reduction in the number of *know* judgements. Recognition responses that are initially assigned a remember judgement are later on experienced with a *know*-type awareness because of the loss of episodic detail, i.e. a “remember-to-know” shift occurs (Conway, Gardiner, Perfect, Anderson, & Cohen, 1997; Dudukovic & Knowlton, 2006; Herbert & Burt, 2003, 2004; Knowlton & Squire, 1995). If at shorter delays depressed participants initially report fewer ‘remember’ judgements, at longer delays when the episodic details are lost and remembering becomes knowing, they would be expected to report fewer ‘know’ judgements. Consequently, at long intervals the recollection deficit associated with dysphoric mood can be expected to be reflected in know judgements.

We also explored whether biasing the processing of pictures with an emotional or neutral interpretation affected memory. Picture processing was biased with the use of picture captions that participants were asked to read and evaluate when viewing the picture (Teasdale et al., 1999). For example, an image of a staircase could be paired with the caption ‘where Johnny had a bad fall’ (negative context), whereas the same image of a staircase could be paired with the caption ‘where the telephone was usually kept’ (neutral context). This procedure has the considerable advantage that basic picture processing is common to the neutral and negative conditions: the same image could be biased across participants with either a neutral or negative caption. An enhancement of picture memory embedded in negative captions was expected due to the memory enhancing effect of emotion.

### 2.1 Method

#### 2.1.1 Participants

Participants were recruited from the volunteer panel of the Medical Research Council Cognition and Brain Sciences Unit. Mood was assessed using the Beck Depression Inventory (BDI), a 21-item self-report measure of depression (Beck, Ward, Mendelson, Mock, & Erbaugh, 1961). The recommended cut-off point for mild depression in the BDI is 9/10 (Kendall, Hollon, Beck, Hammen, & Ingram, 1987). Fourteen subclinically depressed participants with a BDI score of 10 or above and 14 control participants with a BDI score of 9 or below participated; the two groups were matched for age and IQ (Scale 2, Form A of the Cattell Culture Fair Intelligence Test, Cattell & Cattell, 1973). Table 1 summarises the profiles of the 2 groups.

#### 2.1.2 Design and material

Eighty pictures were selected from the International Affective Picture System (IAPS: Lang, Bradley, & Cuthberg, 1997) and other sources where necessary. All pictures were of neutral valence and medium levels of arousal. Using 9-point scales, pilot ratings resulted in an average valence of 5.19 (SD = 0.76) and average arousal of 4.58 (SD = 0.66). The pictures were divided into 4 lists of 20 pictures (A, B, C, and D) that were matched for image content, valence and arousal.

At encoding, pictures from two of the four matched lists were presented in a random order. Negative captions were paired with the pictures in one list, and neutral captions were paired with the pictures in the other. Using the example above, the image of a staircase in one list was paired with a negative caption, whereas a similar but not identical image of a staircase in second list was paired with a neutral caption. All captions were matched for numbers of words. Using 9-point scales, pilot ratings resulted in an average valence of 2.56 (SD = 0.63) and average arousal of 6.44 (SD = 0.84) for the negative captions and in an average valence of 4.97 (SD = 0.48) and average arousal of 3.16 (SD = 0.75) for the neutral captions. At test, the pictures from the remaining two unviewed lists were used as foils. Thus, eighty pictures from the four lists (two viewed lists and two unviewed lists) were presented in a random order. Encoding and test list assignment was randomly determined.

To ensure that attention was paid to the meaning given to the picture by the caption at encoding, participants were required to make an incompatibility judgment, indicating whether the caption was incompatible with the picture it was paired with. An additional 10 neutral filler pictures with incompatible captions (e.g., a picture of some cows in a field would be paired with the incompatible caption: “She washed up after the evening meal”) were interspersed throughout the 40 encoding pictures. Ten filler pictures/caption pairs were also placed at both the beginning and end of the encoding list as buffers; for two of these, the captions were incompatible.

#### 2.1.3 Procedure

Each participant was tested individually. In the encoding phase, neutral pictures were presented one at a time with either a negative or neutral caption presented below each image. Participants indicated when they considered the image-caption pair to be incompatible by pressing the space bar. They were not told that their memory for the pictures would be tested later. The image-caption pair appeared on the screen for 6000 ms with an inter-stimulus interval of 1500 ms. Participants were required to respond within this time frame.

The test phase took place approximately two weeks later (14.7 days +/− 2.4 days). Before presentation of the picture sequence, participants were told that some of the pictures were from the image set presented during the encoding phase two weeks earlier, whereas others were new ones. For each image, participants were asked to indicate whether they recognised the image as one of those presented in the encoding phase. They were given extensive instructions (see Gardiner, Ramponi, & Richardson-Klavehn, 1998) on how to make remember and know judgments following a positive recognition. Participants were instructed to give a remember or know judgment only when they were certain that they recognised the image as one of those belonging to the image set presented at encoding. They were strongly discouraged from guessing: if they were uncertain that an image was an ‘old’ one, they were instructed to say that the image was new. Participants indicated their response by pressing labelled keys on the computer keyboard. Prior to presentation of the test pictures, participants' accuracy in assigning remember and know judgments was assessed with viewed and not-viewed filler pictures in a short practice session in which they explained their reason for a given recognition judgment and the experimenter verified that this response was appropriate.

### 2.2 Results

Mean proportions of picture recognition and of pictures assigned *remember* and *know* judgments, with corresponding judgments to unviewed pictures (i.e. false alarms), are shown in Table 2. An alpha level of .05 was used for all statistical tests in this paper.

#### 2.2.1 Recognition memory

In a 2 (group: subclinically depressed vs. controls) × 2 (image caption: negative vs. neutral) mixed ANOVA on recognition memory corrected for false alarms (i.e. overall recognition proportions minus overall false alarm proportions, for each participant), there was a significant main effect of group, *F* (1,26) = 4.87, *MSE* = .033, *p* < .05*, η*_*p*_^*2*^ = .16, indicating that subclinically depressed participants recognised fewer pictures than controls; thus dysphoric mood appears to also affect memory for pictorial material. Neither the image caption effect, *F* (1,26) = .23, *MSE* = *.009, η*_*p*_^*2*^ = .01*,* nor the interaction of group with caption, *F* (1,26) = .04, *MSE* = *.*009*, η*_*p*_^*2*^ = .00 was significant. A measure of discriminability (*d′; d′* = Z(hits) − Z(false alarm), see Table 3) between the old and new pictures showed exactly the same pattern: *d*′ measures for the subclinically depressed group were significantly lower than those for the control group, *F* (1,26) = 7.25, *MSE* = *.*356, *p* < .*05, η*_*p*_^*2*^ = .22 and neither the image caption effect nor the group by caption interaction were significant (all *F*s < 1).

A measure of criterion (*c)* or of the propensity of participants to produce a positive recognition response was also computed (*c* = (Z(hits) + Z(false alarms)) ⁎ − 0.5, see Table 3). In the corresponding ANOVA a group effect was observed on this measure, *F* (1,26) = 18.07, *MSE* = *.*126, *p* < .05, η_*p*_^*2*^ = *.*41*.* The image caption effect and the interaction were not significant (all *F*s < 1). Thus, relative to controls subclinically depressed participants demonstrated a greater propensity, or bias, to report recognising a picture, as reflected in the larger number of false alarms (see Table 2) reported by the subclinically depressed participants *t* (26) = 4.55, *SEM* = .040, *p* < .05.

#### 2.2.2 Recollection and familiarity

Remember and know judgments, corrected respectively for remember and know false alarms, were also analysed. In the corresponding 2 (group) × 2 (image caption) mixed ANOVA for the remember judgments, neither the main effects nor the interaction were significant (all *F*s < 1). For the know judgments, in the corresponding ANOVA, the group effect approached significance, *F* (1,26) = 3.32, *MSE* = .038*, p* = .08*, η*_*p*_^*2*^ = *.11*, but neither the effect of image caption nor the interaction was significant (all *F*s < 1*)*.

The underlying assumption of the remember/know procedure is that the relationship of recollection and familiarity is a *redundant* one (Joordens & Merikle, 1993): all items are familiar and a subset of these is recollected. An alternative approach to the separation of recollection and familiarity is based on the assumption that the recollection and familiarity processes are *independent*, i.e. some items can only be recollected and some items can only be familiar but some items can be both recollected and familiar (Jacoby, Toth, & Yonelinas, 1993). We derived *recollection* and *familiarity estimates* (Table 3) from the remember and know judgements within a Dual-Process Signal-Detection Model (Yonelinas, Kroll, Dobbins, Lazzara, & Knight, 1998) based on the assumption of independence. This model specifically takes into account response bias, when there is a difference in the number of false alarms between two groups. The formula used to calculate *recollection estimates* from remember and know judgements is reported in Yonelinas et al. (1998). In this model, familiarity, unlike recollection, is assumed to reflect a signal-detection process, so familiarity is measured with *d′*.

For the *recollection estimates*, our results paralleled those reported for the remember judgments, with no effects of caption, group or their interaction (all *F*s < 1). The same analysis on *familiarity d′* showed a significant group effect, *F* (1,26) *= 4.81,* MSE = *.44, p* < .*05, η*_*p*_^*2*^ = .16, indicating that relative to controls, subclinically depressed participants are impaired at discriminating old from new pictures on the basis of familiarity. With this measure no other effects were significant (all *F*s < 1).

### 2.3 Discussion

In Experiment 1 the subclinically depressed participants showed an impairment in recognition memory for pictorial material. They were less able to discriminate between older and newer pictures than control participants implying that dysphoric mood can have a considerable effect on the processing of pictures despite these conveying a richer set of information. Thus, there is no evidence that visual processing, in contrast to verbal processing, is differently affected in depression in the extent to which it can support mnemonic processes. The elevated number of false alarms in subclinically depressed participants relative to controls was also notable; subclinically depressed participants had an increased tendency to judge a picture as seen before at the expense of recognition accuracy.

The recognition deficit in this sample of subclinically depressed participants was captured by a reduction in the number of the know judgements. This result can be nevertheless consistent with the recollection deficit found for verbal material (Drakeford et al., 2010; Jermann et al., 2005; Hertel & Milan, 1994; MacQueen et al., 2002, 2003; Ramponi et al., 2004) when considered in the context of the remember-to-know shift (Conway et al., 1997; Dudukovic & Knowlton, 2006; Herbert & Burt, 2003, 2004; Knowlton & Squire, 1995). In the current study recognition memory was not tested immediately as in the previous studies, but two weeks later to avoid ceiling effects, and what would have been fewer remember judgements at short delays become fewer know judgements at longer delays due to episodic details having been lost.

Finally, the processing of neutral pictures appeared unaffected by negative captions designed to bias picture elaboration. A memory enhancement for the pictures associated with negative captions was not observed. In Experiment 2, negative and neutral emotionality was manipulated by varying inherent picture content.

## 3 Experiment 2

Emotional experience is often described as having two orthogonal dimensions: a valence dimension that characterises the pleasantness or unpleasantness of the experience, and an arousal dimension that characterises how calming or exciting an experience is (Russell, 1980). There is extensive evidence (e.g. Bradley, Greenwald, Petry, & Lang, 1992) that arousal plays a key role in enhancing memory for emotional material. The general finding has been that arousal levels associated with experimental stimuli modulate amygdala activation, which in turn has a role in memory consolidation (see Kensinger, 2004; Kensinger & Corkin, 2004; LaBar & Cabeza, 2006). Recent research in our laboratory (Croucher, 2006; Ewbank, Barnard, Croucher, Ramponi & Calder, 2009; Murphy, Hill, Ramponi, Calder, & Barnard, in press) has studied the “impact” of pictures on an observer; this factor is believed to have a critical role in the enhancement of memory. The term “impact” is used in visual media to describe particularly eye-catching pictures. The degree of affective impact reflects the extent to which a picture personally affects the viewer and how quickly the viewer grasps a picture's content. Distinct stimuli can be appraised impersonally and “propositionally” as having comparable negative emotional content even though they may not give rise to comparable levels of genuinely felt affect (Teasdale & Barnard, 1993). Croucher (2006) found that the degree of affective impact modulated recollection of pictures in a recognition task: negative pictures of high impact were better remembered than negative pictures of low impact, even though these image sets were matched for negative valence and arousal. Murphy et al. (in press) found that high impact negative pictures compared to matched low impact negative and neutral pictures receive priority processing in visual attention, and Ewbank et al. (2009) found increases in amygdala activation when viewing high impact relative to low impact negative pictures and yet no difference when viewing low impact negative pictures relative to neutral ones despite a difference in valence.

The second experiment examined the effect of dysphoric mood on recollection and familiarity for pictures of inherent negative emotional content. Subclinically depressed and control participants reported remember and know judgements after viewing neutral pictures and two sets of pictures that varied in affective impact but were matched on other key dimensions that could influence retention (arousal, valence, distinctiveness, approach/avoidance and visual complexity). In order to achieve a retention interval more comparable to that employed in studies of the influence of mood on recollection and familiarity for words whilst avoiding ceiling effects, we reduced exposure time and made use of an incidental monitoring task that did not require aspects of picture content to be actively processed.

A replication of the memory deficit observed in Experiment 1 in subclinically depressed participants would confirm that dysphoric mood influences memory for pictorial material and verbal material similarly. We predicted that the recollection deficit would be captured by the remember judgements as previously found for verbal material due to the similarly short gap between encoding and test. We expected that the negative pictures of high affective impact would be better remembered than both the low affective impact and neutral pictures.

### 3.1 Method

#### 3.1.1 Participants

Participants were other participants recruited from the volunteer panel of the Cognition and Brain Sciences Unit. Twenty subclinically depressed participants with BDI scores of 10 or above were matched for age and IQ with twenty control participants with BDI scores of 9 or below. Table 4 summarises the profiles of the 2 groups.

#### 3.1.2 Design and material

The negatively-valenced pictures were selected from a set of IAPS pictures rated for valence, arousal, distinctiveness, approach/avoidance and visual complexity by Croucher (2006). The impact scale varied from 9 (indicating maximum affective impact) to 0 (indicating minimum affective impact). Sixty negatively-valenced pictures were used, with half of the pictures of high impact (*M* = 7.13, *SD* = 0.93) and the other half of low impact (*M* = 3.67, *SD* = 0.53). The two sets were matched for valence (*M* = 2.01, *SD* = 0.73; *t* (58) = 1.16, *SEM* = .187, *d* = .30), arousal (*M* = 4.76, *SD* = 1.17; *t* (58) = .38, *SEM* = .304, *d* = .10), distinctiveness (*M* = 5.80, *SD* = 1.19; *t* (58) = 1.09, *SEM* = .306, *d* = .29), approach/avoidance (*M* = 7.30, *SD* = 1.03; *t*(58) = .12 , *SEM* = .268, *d* = .03) and visual complexity (*M* = 4.12, *SD* = 1.09; *t*(58) = 1.05, *SEM* = .282, *d* = .29). There were also 30 pictures of neutral valence (*M* = 5.23, *SD* = 0.64) and arousal (*M* = 4.79, *SD* = 0.73): half of the neutral pictures were from the IAPS and the other half were drawn from other sources to allow them to be matched on arousal with the negatively-valenced pictures. The 30 pictures in each of the high impact, low impact and neutral sets were divided into two lists matched for each of the criteria listed above. At encoding, participants were shown one of the lists for each set such that 45 pictures were presented overall. At test, pictures from the second (unviewed) list from each set acted as foils. Encoding list assignment was randomly determined.

In order to ensure attention was directed at the pictures at encoding without requiring detailed attention to picture content, participants were asked to press the spacebar to pictures that appeared upside down. To this end, 15 filler pictures were presented in an inverted orientation. Fifteen filler pictures were also placed at the beginning and end of the testing pictures as buffers and two of these were also presented upside down. Fifteen additional fillers were presented interspersed with the 45 critical pictures to increase the numbers of pictures presented. At study and test pictures were presented in a pseudorandom order so that no more than 2 pictures of the same type were presented in sequence.

#### 3.1.3 Procedure

Each participant was tested individually. Participants were not told that their memory for the pictures would be tested later and were asked to judge whether an image was presented upside down. A fixation cross appeared for 500 ms and after an additional 500 ms, the image appeared for 1000 ms. Forty-five minutes later participants' recognition for the pictures was tested. The same procedure as Experiment 1 was followed for the recognition decision and remember/know judgments task.

### 3.2 Results

Mean picture recognition scores and the proportions of pictures assigned *remember* and *know* judgments, with respective false alarms, are shown in Table 5. Signal detection measures, (*d′* and *c*), and *recollection* and *familiarity* estimates are reported in Table 6.

#### 3.2.1 Recognition memory

In a 2 (group: subclinically depressed and controls) × 3 (picture-type: high impact, low impact and neutral) repeated-measures ANOVA of recognition scores corrected for false alarms, the effect of picture-type was significant, *F*(2,76) *=* 24.215, *MSE =* .017, *p <* .001, *η*_*p*_^*2*^ *=* .39; neither the effect of group nor its interaction with picture-type was significant (all *F*s < 1). Planned comparisons showed that participants recognised more high impact than low impact pictures, *t*(39) *=* 3.77, *SEM =* .021, *p <* .001, *d =* .60, and more low impact than neutral pictures, *t*(39) *=* 3.56, *SEM =* .035, *p <* .001, *d =* .56.

The corresponding ANOVA on *d′* (see Table 6) mirrors these results with a main effect of picture-type, *F*(2,76) *=* 12.306, *MSE =* .540, *p <* .001, *η*_*p*_^*2*^ *=* .24 , but no main effect of group, *F*(1,38) *=* 2.579, *MSE =* 1.454, *η*_*p*_^*2*^ *=* .06, or interaction, *F*(2,76) *=* .824, *MSE =* .540, *η*_*p*_^*2*^ *=* .02. In the corresponding ANOVA on the criterion measures there was a significant effect of picture-type, *F*(2,76) *=* 8.358, *MSE =* .089, *p =* .001, *η*_*p*_^*2*^ *=* .18, and a picture-type by group interaction, *F*(2,76) *=* 3.356, *MSE =* .089, *p <* .05, *η*_*p*_^*2*^ *=* .08. The interaction indicated that the subclinically depressed participants adopted a more liberal criterion as in Experiment 1, than the controls when recognising the neutral pictures *(t*(38) *=* 2.17, *SEM =* .135, *p <* .05, *d =* .80) but not when recognising the high impact *(t*(38) *=* .34, *SEM =* .154, *d =* .11) or low impact pictures (*t*(38) *=* .89, *SEM =* .150, *d =* .18).

#### 3.2.2 Recollection and familiarity

For the remember judgments corrected for false alarms, the same 2 (group) × 3 (picture-type) ANOVA showed that the group effect was significant, *F*(1,38) *=* 4.874, *MSE =* .080, *p <* .05, *η*_*p*_^*2*^ *=* .1, with the subclinically depressed participants remembering fewer pictures than controls. The picture-type effect was also significant, *F*(2,76) *=* 32.450, *MSE =* .017, *p <* .001, *η*_*p*_^*2*^ *=* .46, whereas the group by picture-type interaction was not, *F*(2,76) *=* .895, *MSE =* .018, *η*_*p*_^*2*^ *=* .02. The same results were observed in the corresponding ANOVA for the *recollection estimates* (reported in Table 6) where a significant group effect, *F*(1,38) *=* 4.132, *MSE =* .087, *p =* .05, *η*_*p*_^*2*^ *=* .10, and picture-type effect, *F*(2,76) *=* 35.950, *MSE =* .017, *p <* .001, *η*_*p*_^*2*^ *=* .49, were observed, but the interaction was not significant, *F*(2,76) *=* .947, *MSE =* .017, *η*_*p*_^*2*^ *=* .02. In both groups, the high impact pictures were associated with more remember judgements than the low impact pictures, control: *t*(19) *=* 3.61, *SEM =* .041, *p <* .05, *d =* .81; subclinically depressed: *t*(19) *=* 2.11, *SEM =* .041, *p <* .05, *d =* .47, and were associated with higher *recollection estimates*, control: *t*(19) *=* 3.79, *SEM =* .040, *p <* .05, *d =* .85; subclinically depressed: *t*(19) *=* 2.42, *SEM =* .039, *p <* .05, *d =* .53; in planned comparisons the low impact pictures were associated with more remember judgements than the neutral pictures (Control: *t*(19) *=* 6.02, *SEM =* .044, *p <* .05, *d =* .68; subclinically depressed: *t*(19) *=* 2.41, *SEM =* .048, *p <* .05, *d =* .54) and with higher *recollection estimates* (Control: *t*(19) *=* 3.39, *SEM =* .040, *p <* .05, *d =* .76; subclinically depressed: *t*(19) *=* 2.35, *SEM =* .049, *p <* .05, *d =* .53). The recollection deficit observed in subclinically depressed participants was not attenuated for the negative pictures, as confirmed by the absence of a group by picture-type interaction

For the corrected know judgments, in the corresponding ANOVA we did not find a significant effect of group, *F*(1,38) *=* 4.113, *MSE =* .062, *η*_*p*_^*2*^ *=* .07, picture type, *F*(2,76) *=* 2.119, *MSE =* .009, *η*_*p*_^*2*^ *=* .05, or an interaction *F*(2,76) *=* .683, *MSE =* .009, *η*_*p*_^*2*^ *=* .01). With the *familiarity d′* estimates we observed no effect of group, (1,38) *=* .336, *MSE =* 1.116, *η*_*p*_^*2*^ *=* .009, and no significant interaction, *F*(2,76) *=* .129, *MSE =* .300, *η*_*p*_^*2*^ *=* .003), but in this case the effect of picture type, *F*(2,76) *=* 6.157, *MSE =* .300, *p <* .05, *η*_*p*_^*2*^ *=* .14, was significant. The neutral pictures were less *familiar* than the low impact, *t*(39) *=* 3.05, *SEM =* .124, *p <* .05, *d =* .43, and high impact pictures, *t*(39) *=* 2.78, *SEM =* .131, *p <* .05, *d =* .48, but the high and low impact pictures did not differ in how familiar they were, *t*(39) *=* .134, *SEM =* .106, *d =* .02.

### 3.3 Discussion

While a global difference in recognition memory between the subclinically depressed and control groups was not observed, a deficit was detected specifically in the recollection component. Subclinically depressed participants reported fewer recognition responses accompanied by remember judgements than control participants. These results replicate previous findings for recollection with verbal material (Drakeford et al., 2010; Hertel & Milan, 1994; Jermann et al., 2005; MacQueen et al., 2002; 2003; Ramponi et al., 2004) and extend those findings to pictures. Dysphoric mood particularly affects memory for the episodic details accompanying events, rather than affecting memory for the event *per se* (Ramponi, Nayagam, & Barnard, 2009; Raes et al., 2006) and we can now conclude, on the basis of this current evidence, that this holds for pictures. Again, as in Experiment 1, the visual processes engaged to encode and retain (particularly context) information presented in pictorial form are clearly affected even in subclinical forms of depression.

Negatively-valenced pictures were better recognised and recollected than neutral pictures with negative pictures of high affective impact being better recollected, replicating Croucher (2006). With the familiarity estimates, but not with the know judgements, we found that for all participants negative pictures (whether of high or low impact) were judged to be more familiar than the neutral ones.

## 4 General discussion

In this study we investigated how dysphoric mood affects recollection and familiarity for pictorial material that varies in emotionality as determined by verbal description or inherent content. We conclude that recognition memory for pictorial material is adversely affected by mood. Visual processing to the extent that it supports the encoding, elaboration and retention of information in pictorial form is also impaired by dysphoric mood.

In relation to recollection and familiarity, our results are largely consistent with the view that recollection of the episodic context is impaired by dysphoric mood, (Drakeford et al., 2010; Hertel & Milan, 1994; Jermann et al., 2005; MacQueen et al., 2002; 2003; Ramponi et al., 2004, but see Jermann et al., 2009). In Experiment 1, recognition memory for the neutral pictures was significantly reduced in subclinically depressed participants compared to controls. This reduction was captured by the familiarity component of recognition memory, when memory is consolidated and a remember-to-know shift has occurred due to contextual details having been lost (Conway et al., 1997; Dudukovic & Knowlton, 2006; Knowlton & Squire, 1995), the original deficit in recollection can subsequently be reflected in recognition memory experienced as knowing. In Experiment 2, where recognition memory was tested soon after pictures were viewed, a marked deficit in recollective experience, but not in familiarity, was observed in the subclinically depressed participants.

A notable finding from both experiments involves criterion differences between the subclinically depressed and control groups. In Experiment 1, subclinically depressed participants had twice as many false alarms as control participants, showing that subclinically depressed participants adopted a very liberal criterion when deciding whether or not they had seen a neutral picture irrespective of the encoding context. As indicated by the discrimination measure (*d′*), subclinically depressed participants were less able to discriminate between old and new pictures and, as indicated by the criterion measure (*c*), they opted for a more inclusive strategy, thus risking more false positive judgements. A similar pattern was obtained in Experiment 2, but only with the pictures conveying neutral content. No reliable differences on criterion measures were obtained with the negative pictures.

It is possible that under considerable uncertainty, created by long retention intervals and/or indistinctive pictures of neutral content, subclinically depressed participants' negative appraisal of their own ability to remember events may contribute to a choice of an over-inclusive strategy. Previous findings reported in the recognition memory literature concerning criteria differences for dysphoric mood have shown very little consistency. Some studies have reported a more liberal criterion for negative words related to dysphoric mood (Deijen, Orlebeke, & Rijsdijk, 1993; Zuroff, Colussy, & Wieglus, 1983), while other studies have reported a more conservative criterion (Corwin, Peselow, Feenan, Rotrosen, & Fieve, 1990; Dunbar & Lishman, 1984) and yet others do not report any criterion shift (Brebion, Smith, & Widlocher, 1997; Channon, Baker, & Robertson, 1993; Watts, Morris, & Macleod, 1987). A number of methodological factors may be responsible for these differences, including differences in task demands, materials and severity of mood. The general conclusion is that, at a minimum, accurate interpretation of results must consider these biases (e.g., Zuroff et al., 1983).

The presence of a marked liberal criterion with neutral pictures at two different delays and for both low (Experiment 1) and high levels of recollection (Experiment 2) appears consistent with differences linked to dysphoric mood in the problem-solving literature (Slife & Weaver, 1992). One difference can be characterised as a pure cognitive deficit (represented by the discrimination measure *d′*) and the other difference can be characterised as a difference in metacognitive processes involved in strategy selection (as represented by the criterion measure *c*). This analogy could help to resolve earlier empirical discrepancies. For example, in discussing the theoretical basis for impaired initiative in depressed states, Hertel and Hardin (1990) identified motivational and metacognitive factors as plausible explanatory candidates. This hypothesis of differences in metacognitive processes related to mood when recognising neutral pictures could be further explored by manipulating the “expectancy” of the likelihood that old pictures would appear in the recognition test (see Gardiner, Richardson-Klavehn, & Ramponi, 1997; McCabe & Balota, 2007) and by measuring participants' self-perception of their mnemonic competence. Criterion differences, and by implication their metacognitive origins, have very notable effects on remember and know judgements (Donaldson, 1996; Gardiner, Ramponi, & Richardson-Klavehn, 2002; Parks & Yonelinas, 2007; Postma, 1999; Wixted, 2007) that could substantially alter the view presented thus far on the effect of mood on memory; hence, these warrant systematic future investigation.

While the neutral and negative captions of Experiment 1 did not have an observable effect on the recognition memory for either control or subclinically depressed groups, a mnemonic advantage for negatively valenced pictures was observed in Experiment 2. As anticipated, this effect was particularly marked for the recollection of pictures of high relative to low affective impact that were equal in rated arousal and valence. This result replicates the finding that the emotion effect is captured by the recollection component (Comblain et al., 2004; Croucher, 2006; Dahl et al., 2006; Dolcos et al., 2005; Ochsner, 2000; Ritchey, Dolcos, & Cabeza, 2008; Sharot et al., 2004; Sharot & Yonelinas, 2008).

The recollection deficit observed in subclinically depressed participants was not reduced for the negative pictures, and thus, a mood-congruent-memory effect was not observed. Mood-congruent-memory effects have been observed primarily with self-referent encoding or autobiographical elaboration (Bradley & Mathews, 1983; Lewis et al., 2005; Matt, Vazquez, & Campbell, 1992). Impact ratings reflected the extent to which an image had particular emotional impact and salience to the self, but not to which it was inherently autobiographical. It may be far easier for subclinically depressed individuals to relate words of negative valence, like “regret” or “sorrow”, to their personal experience (Kensinger, 2004) than to relate pictures that depict specific events that are happening to someone else. Furthermore, in studies of recognition memory for emotional faces, mood-congruence effects have sometimes been observed (Gilboa-Schechtman et al., 2002; Jermann et al., 2008; Ridout et al., 2003; Ridout, Noreen, et al., 2009) and sometimes not (e.g. Ridout, Dritschel, et al., 2009). Ridout, Dritschel, et al. (2009) found that mood-congruent-memory was absent in participants that were not oriented towards the affective element of the faces at encoding. The task that participants were asked to carry out at encoding in the current experiments simply ensured attention to the pictures. In one case, they reported whether the caption was coherent with the picture and in the other case, whether the picture was inverted. At encoding, participants were not explicitly oriented to appraise the picture's affective component, a factor that may explain why a mood-congruent effect was not observed. Nevertheless, the intrinsically negative pictures were still better recollected, probably indicating that more semantic elaboration had occurred at encoding, but the semantic elaboration for negative pictures was not more extensive in the subclinically depressed group.

Finally, in relation to mood-congruent memory, one caveat of this study is that our results rely on normative valence and arousal ratings of the negative and neutral pictures. Subjective ratings were not collected, so it is possible that mood-congruent effects had occurred but these would only have been apparent if subjective ratings were considered. A second caveat of this study is that the effect of subclinical depression on recognition memory for the negative and neutral pictures was not measured at longer time intervals to test the patterns of the remember-to-know shift. This is also important in view of evidence that the effect of emotion on memory can differ with time (e.g. Sharot & Yonelinas, 2008). Ritchey et al. (2008) provide some evidence that, over a time interval of a week, recollection for emotionally negative pictures increased even though overall recognition memory remained similar. If this is the case, it is possible that remember-to-know shifts may have different time courses for pictures of negative content relative to neutral pictures.

In summary, the current experiments complement and extend prior work with verbal material by showing that dysphoric mood has adverse effects on the recognition of pictures. In Experiment 2 the deficit is confined to the recollection component of recognition memory and the effects observed after a two-week interval in Experiment 2 are consistent with controls remembering more at the outset leading to a greater opportunity than the subclinically depressed participants to report knowing pictures at the longer interval. Along with a core deficit in memory comparable to that observed with verbal material, the presence in subclinical depression of a more liberal criterion with neutral material is consistent with a second, qualitatively distinct difference in metacognition in the subclinically depressed group that was confined to pictures that were unremarkable in their content. Control and subclinically depressed participants showed comparable memory enhancement for negatively valenced pictures.

## Figures and Tables

**Table 1:** Mean scores and SD of individual differences indices and of demographic characteristics for subclinically depressed and control participant.

	Control participants (N = 14)		Subclinically depressed participants	
			(N = 14)	
	*M*	*SD*	*M*	*SD*
BDI	2.79	2.32	14.43	3.84
IQ	118.93	9.69	118.36	24.41
AGE	33.71	11.81	33.57	13.69
Male:Female (N)	7	7	6	8

**Table 2:** Proportions of overall picture recognition and the proportion of remembered and known pictures, with respective false alarms (FA) proportions, for control and subclinically depressed participants as a function of contextual valence.

Captions	Hits						False alarms					
	Recognition		Remember		Know		Recognition		Remember		Know	
	*M*	*SE*	*M*	*SE*	*M*	*SE*	*M*	*SE*	*M*	*SE*	*M*	*SE*
*Control participants (N = 14)*												
Neutral	.63	.04	.32	.04	.31	.04	.16	.02	.05	.01	.10	.01
Negative	.62	.04	.32	.05	.30	.03						

*Subclinically depressed participants (N = 14)*												
Neutral	.71	.02	.36	.05	.35	.04	.34	.04	.11	.02	.23	.03
Negative	.69	.02	.37	.04	.32	.04						

**Table 3:** Proportion of recollected and familiar pictures and measures of discriminability (d′) and criterion (c), for control and subclinically depressed participants as a function of contextual valence.

Captions	Recollection		Familiarity *d′*		*d′*		*c*	
	*M*	*SE*	*M*	*SE*	*M*	*SE*	*M*	*SE*
*Control participants (N = 14)*								
Neutral	.28	.04	1.15	.17	1.44	.16	.34	.08
Negative	.29	.05	1.12	.08	1.39	.12	.37	.07

*Subclinically depressed participants (N = 14)*								
Neutral	.28	.05	.78	.16	1.01	.11	−.06	.06
Negative	.29	.04	.70	.14	.95	.10	.04	.08

**Table 4:** Mean scores and SD of individual differences indices and of demographic characteristics for subclinically depressed and control participants in Experiment 2.

	Control participants (N = 20)		Subclinically depressed participants	
			(N = 20)	
	*M*	*SD*	*M*	*SD*
BDI	3.85	3.30	18.20	7.59
IQ	113.20	7.87	114.15	8.72
AGE	34.75	11.61	35.70	12.65
Male:Female (N)	7	13	6	14

**Table 5:** Proportions of overall picture recognition and the proportion of remembered and known pictures, with respective false alarms (FA) proportions, for control and subclinically depressed participants in Experiment 2 as a function of picture valence.

Pictures	Hits						False alarms					
	Recognition		Remember		Know		Recognition		Remember		Know	
	*M*	*SE*	*M*	*SE*	*M*	*SE*	*M*	*SE*	*M*	*SE*	*M*	*SE*
*Control participants (N = 20)*												
High impact	.82	.03	.76	.04	.06	.02	.03	.02	.02	.01	.01	.01
Low impact	.72	.04	.62	.05	.10	.01	.03	.01	.02	.01	.01	.01
Neutral	.59	.05	.48	.04	.11	.03	.03	.01	.02	.01	.01	.01

*Subclinically depressed participants (N = 20)*												
High impact	.78	.03	.62	.04	.16	.03	.05	.02	.04	.02	.02	.01
Low impact	.72	.04	.52	.05	.20	.04	.05	.02	.03	.01	.02	.01
Neutral	.63	.04	.41	.05	.22	.05	.09	.02	.03	.01	.05	.02

**Table 6:** Proportion of recollected and familiar pictures and measures of discriminability (d′) and criterion (c), for control and subclinically depressed participants as a function of picture valence.

Pictures	Recollection		Familiarity *d′*		*d′*		*c*	
	*M*	*SE*	*M*	*SE*	*M*	*SE*	*M*	*SE*
*Control participants (N = 20)*								
High impact	.76	.04	1.28	.15	3.32	.22	.52	.12
Low impact	.61	.05	1.29	.11	2.78	.18	.74	.10
Neutral	.47	.04	.97	.17	2.45	.22	.97	.08

*Subclinically depressed participants (N = 20)*								
High impact	.60	.04	1.45	.17	2.79	.18	.58	.10
Low impact	.51	.05	1.42	.21	2.66	.23	.65	.11
Neutral	.39	.05	1.01	.18	2.03	.20	.68	.11
